# Functional MRI of the vocalization-processing network in the macaque brain

**DOI:** 10.3389/fnins.2015.00113

**Published:** 2015-04-01

**Authors:** Michael Ortiz-Rios, Paweł Kuśmierek, Iain DeWitt, Denis Archakov, Frederico A. C. Azevedo, Mikko Sams, Iiro P. Jääskeläinen, Georgios A. Keliris, Josef P. Rauschecker

**Affiliations:** ^1^Department of Neuroscience, Georgetown University Medical CenterWashington, DC, USA; ^2^Department of Physiology of Cognitive Processes, Max Planck Institute for Biological CyberneticsTübingen, Germany; ^3^IMPRS for Cognitive and Systems NeuroscienceTübingen, Germany; ^4^Brain and Mind Laboratory, Department of Neuroscience and Biomedical Engineering, Aalto University School of ScienceAalto, Finland; ^5^Bernstein Centre for Computational NeuroscienceTübingen, Germany; ^6^Department of Biomedical Sciences, University of AntwerpWilrijk, Belgium; ^7^Institute for Advanced Study and Department of Neurology, Klinikum Rechts der Isar, Technische Universität MünchenMünchen, Germany

**Keywords:** auditory cortex, monkey, species-specific calls, spectrotemporal features, higher-level representations

## Abstract

Using functional magnetic resonance imaging in awake behaving monkeys we investigated how species-specific vocalizations are represented in auditory and auditory-related regions of the macaque brain. We found clusters of active voxels along the ascending auditory pathway that responded to various types of complex sounds: inferior colliculus (IC), medial geniculate nucleus (MGN), auditory core, belt, and parabelt cortex, and other parts of the superior temporal gyrus (STG) and sulcus (STS). Regions sensitive to monkey calls were most prevalent in the anterior STG, but some clusters were also found in frontal and parietal cortex on the basis of comparisons between responses to calls and environmental sounds. Surprisingly, we found that spectrotemporal control sounds derived from the monkey calls (“scrambled calls”) also activated the parietal and frontal regions. Taken together, our results demonstrate that species-specific vocalizations in rhesus monkeys activate preferentially the auditory ventral stream, and in particular areas of the antero-lateral belt and parabelt.

## Introduction

The concept of two streams in auditory cortical processing, analogous to that in visual cortex (Mishkin et al., 1983), was proposed more than a decade ago (Rauschecker, 1998a; Rauschecker and Tian, 2000). The concept was supported by contrasting patterns of anatomical connections in the macaque from anterior/ventral and posterior/dorsal belt regions of auditory cortex to segregated domains of lateral prefrontal cortex (Romanski et al., 1999) and by different physiological properties of these belt regions. In particular, the anterior lateral belt (area AL) in the macaque exhibited enhanced selectivity for the identity of sounds (monkey vocalizations), whereas the caudal lateral belt (area CL) was particularly selective to sound location (Tian et al., 2001; see also Kuśmierek and Rauschecker, 2014). Evidence for segregated streams of auditory cortical processing has also been provided in human studies (Maeder et al., 2001; Arnott et al., 2004; Ahveninen et al., 2006).

Use of species-specific vocalizations for auditory stimulation in the macaque is of particular interest in the context of the ongoing debate about the evolution of speech and language (Rauschecker, 2012; Bornkessel-Schlesewsky et al., 2015). Comparative approaches have focused on identifying the common neural networks involved in the processing of speech in humans and of vocalizations in non-human primates (Gil-da-Costa et al., 2004; Frey et al., 2008, 2014; Petrides and Pandya, 2009; Joly et al., 2012b). Monkey calls convey semantic information about objects and events in the environment as well as about affective states of individuals, similar to information contained in human communication sounds and speech (Cheney and Seyfarth, 1990; Ghazanfar and Hauser, 1999; Yovel and Belin, 2013). An open question regarding the vocalization-processing network in the macaque brain is whether it also carries information about the motor actions necessary to produce the vocalizations, as has been shown in humans listening to speech and music (Wilson et al., 2004; Leaver et al., 2009).

Several studies have examined the representation of complex sounds, including vocalizations, in the macaque brain using neuroimaging techniques (Poremba et al., 2003; Petkov et al., 2008; Joly et al., 2012b). In particular, the first fMRI study by Petkov et al. (2008) found activation specific to monkey vocalizations in the anterior STG region. One of the aims in later studies has been to characterize the physiological properties of the anterior superior temporal (aSTG) region that shows sensitivity to higher-level spectrotemporal features in vocalizations (Russ et al., 2008; Kikuchi et al., 2010, 2014; Perrodin et al., 2011; Fukushima et al., 2014). A recent comparative study by Joly et al. (2012b) replicated and extended these results by analyzing fMRI images of the entire brain and found an involvement of orbitofrontal cortex in the processing of monkey vocalizations. Given that the ventral pathway continues into orbitofrontal and ventrolateral prefrontal cortex (vlPFC) (Barbas, 1993; Romanski et al., 1999; Cohen et al., 2007; Petkov et al., 2015), this finding is of particular interest.

In humans, the ventral auditory pathway is thought to be particularly involved in the recognition and identification of vocalizations as well as speech (Binder et al., 2000; DeWitt and Rauschecker, 2012). By contrast, the dorsal pathway is involved primarily in processing sound source location and motion in both humans and animals (Maeder et al., 2001; Tian et al., 2001; Arnott et al., 2004). However, a recent proposal, derived from both human and non-human primate studies, suggests that the dorsal stream may also play a role in sensorimotor integration and control of complex sounds, including speech (Rauschecker and Scott, 2009; Rauschecker, 2011). Thus, activation of frontal and parietal regions might also be expected when monkeys are presented with conspecific vocalization sounds.

Here we identified which brain regions of the macaque monkey are sensitive to conspecific vocalizations using whole-brain functional magnetic resonance imaging (fMRI). We found the most distinct activation in the anterior STG and along the auditory ventral stream, but some clusters of activation were also found in prefrontal, premotor, and parietal cortex when comparing monkey vocalizations to environmental sounds. These findings are discussed in terms of their functional significance.

## Materials and methods

### Subjects

Two male rhesus monkeys *(Macaca mulatta*) weighing 10–12 kg participated in our awake-fMRI experiments. Each animal was implanted with an MRI-compatible headpost (Applied Prototype) secured to the skull with ceramic screws (Thomas Recording), plastic strips, and bone cement (Osteobond, Zimmer). All surgical procedures were performed under general anesthesia with isoflurane (1–2%) following pre-anesthetic medication with ketamine (13 mg/kg) and midazolam (0.12 mg/kg). The experiments were approved by the Georgetown University Animal Care and Use Committee and conducted in accordance with standard NIH guidelines.

### Behavioral training

To ensure the monkeys attended to each stimulus for which a brain volume was acquired, we adapted a go/no-go auditory discrimination task (Kuśmierek and Rauschecker, 2009; Kikuchi et al., 2010) for sparse-sampling functional MRI.

First, each monkey was trained to lie in sphinx position in an MRI-compatible primate chair (Applied Prototype) placed inside a double-walled acoustic chamber simulating the scanner environment. Inside the chamber, the animals were trained to be accustomed to wearing headphone equipment and hearing (simulated) scanner noise, presented by a loudspeaker. Eye movements were monitored using an infrared eye-tracking system (ISCAN). Analog output of the tracker was sampled with an analog-to-digital conversion device (National Instruments). A PC running Presentation software (Neurobehavioral Systems) was used to present visual and auditory stimuli, control the reward system, and trigger imaging data acquisition (see below).

**Figure 1:**
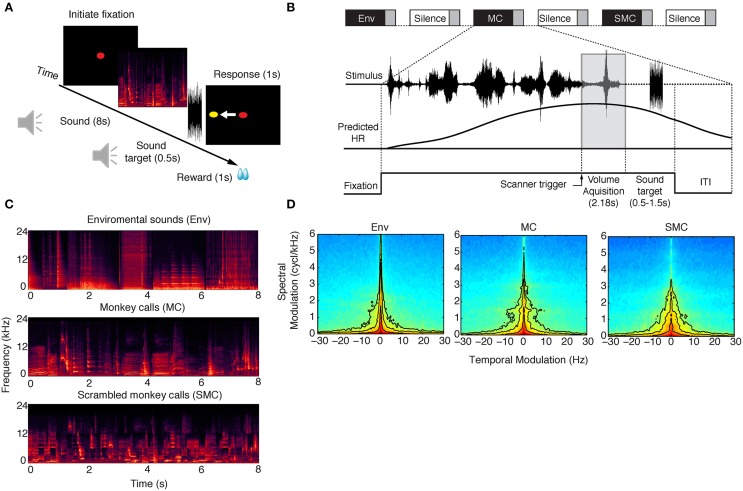
**Behavioral paradigm and example stimuli for each sound category. (A)** The monkey had to keep fixation on a central spot while stimuli were presented from one of three sound categories: environmental sounds (Env), monkey calls (MC) and scrambled monkey calls (SMC). Next, a target sound (white noise, 500 ms) was played after a random delay of 0.5–1.5 s at the end of each stimulus period, and the animals were required to make a saccade to an imaginary cue position (yellow cue). The imaginary target was chosen to be either on the left or the right side of the screen, and the animal was instructed at the beginning of each session where the target was going to appear (see Materials and Methods). **(B)** All conditions were presented in a sparse-sampling design to avoid interference between the hemodynamic response (HR) generated by the scanner noise and by the stimuli. The inter-trial interval (ITI) lasted for 2 s, and the monkey was then allowed to start a new trial by initiating fixation once again. **(C)** Eight-second series of spectrograms from the three sound categories presented. **(D)** Average modulation spectra for each stimulus category. Pearson correlations between average modulation spectra were: MC vs. SMC = 0.92, MC vs. Env = 0.86.

After the animal completed the fixation training, a go/no-go auditory discrimination task was introduced, in which the monkeys could initiate a trial by holding fixation on a central red spot while a block of auditory stimuli would be simultaneously presented. After the first 6 s of auditory stimulation, a trigger was sent to the scanner, starting the acquisition of an image volume (Figure 1B). Following acquisition and a random delay, the target sound (white noise) was presented, cueing a saccade to the left or to the right side as signaled at the beginning of each experimental session (Figure 1A). To provide feedback, after the response window, a yellow spot was shown indicating the correct target location. Finally, contingent on performance, the animal received a juice reward. An inter-trial interval of at least 2 s was enforced before the next trial could be initiated by fixation. Every sound presentation trial was followed by a “silence” trial, allowing for measurement of baseline blood oxygen level dependent (BOLD) signal. Monkey 1 (M1) performed the task correctly for over 90% of the trials. Monkey 2 (M2) was not able to perform the saccadic go/no-go discrimination task with high accuracy and was therefore scanned while passively listening to the acoustic stimuli. To ensure stable attention, M2 was rewarded for successfully holding fixation throughout the trial.

### Auditory stimuli

Three sound categories were used in the experiments: environmental sounds (Env), monkey vocalizations or calls (MC), and scrambled monkey calls (SMC). Spectrograms of example clips from each of these three categories are illustrated in Figure 1C. Environmental sounds were obtained from multiple online sources and from recordings made in our laboratory facilities (Kuśmierek and Rauschecker, 2009). They included the sounds of vehicles, cages, water, food containers, clocks, cameras, applause, coins, footsteps, chewing, heartbeats, horns, and telephones (*n* = 56). The mean duration of the Env stimuli was 1.14 s (range: 0.96–2.6 s). Monkey calls were obtained from recordings made outside our colony [M. Hauser and/or Laboratory of Neuropsychology (LN) library]. Monkey vocalizations (*n* = 63) consisted of grunts, barks, warbles, coos, and screams, as used in prior studies (Rauschecker et al., 1995; Tian et al., 2001; Kuśmierek et al., 2012). The mean duration of the vocalization stimuli was 0.67 s (range: 0.13–2.34 s). SMC were generated by randomly rearranging 200 ms by 1-octave tiles of the constant-Q spectrogram (Brown, 1991) for each monkey call and reconstructing a time-domain waveform with an inverse transform (Schörkhuber and Klapuri, 2010). Transposition along the time axis was not constrained while transposition along the frequency axis was restricted to displacement by a single octave. For each trial, a random selection of stimuli from one class (MC, Env, or SMC) was arranged sequentially into a smooth auditory clip that lasted for the duration of the trial (8 s).

Sounds were presented through modified electrostatic in-ear headphones (SRS-005S + SRM-252S, STAX), mounted on ear-mold impressions of each animal's pinna (Sarkey Eden Prairie) and covered with a custom-made earmuff system for sound attenuation. To match loudness, the stimuli were played through the sound presentation system and re-recorded with a probe microphone (Brüel and Kjær, type 4182 SPL meter) inserted in the ear-mold of an anesthetized monkey. The recordings were then filtered with an inverted macaque audiogram (Jackson et al., 1999) to simulate the effect of different ear sensitivity at different frequencies, analogous to the dB(A) scale for humans. The stimuli were finally equalized so that they produced equal maximum root mean square (RMS) amplitude (using a 200-ms sliding window) in filtered recordings (Kuśmierek and Rauschecker, 2009). During experiments, all stimuli were amplified (Yamaha AX-496) and delivered at a calibrated RMS amplitude of ~80 dB SPL.

### Analyses of sound categories

A modulation spectrum analysis (Singh and Theunissen, 2003) was performed for each sound with the STRFpak Matlab toolbox (http://strfpak.berkeley.edu). We obtained a spectrogram of each sound by decomposing it into frequency bands using a bank of Gaussian filters (244 bands, filter width = 125 Hz). The filters were evenly spaced on the frequency axis (64–48,000 Hz) and separated from each other by one standard deviation. The decomposition resulted in a set of narrow-band signals, which were then cross-correlated with each other and themselves to yield a cross-correlation matrix. This matrix was calculated for time delays of ±150 ms, and the two-dimensional Fourier transform of this matrix was calculated to obtain the modulation spectrum of each sound (Figure 1D).

### Data acquisition

Images were acquired with a horizontal MAGNETOM Trio 3-T scanner (Siemens) with a 60-cm bore diameter. A 12-cm custom-made saddle shape radiofrequency coil (Windmiller Kolster Scientific) covered the entire brain and was optimized for imaging the temporal lobe. The time series consisted of gradient-echo echo-planar (GE-EPI) whole-brain images obtained in a sparse acquisition design. Sparse sampling allows single volumes to be recorded coincidentally with the predicted peak of the evoked hemodynamic response (Hall et al., 1999). This helps to avoid contamination of the measured stimulus-specific BOLD response by the scanner-noise-evoked BOLD response. Further, by triggering acquisition 6 s after stimulus onset, the auditory stimulus was presented without acoustic interference from gradient-switching noise, typical of a continuous fMRI design. For the functional data, individual volumes with 25 ordinal slices were acquired with an interleaved single-shot GE-EPI sequence (TE = 34 ms, TA = 2.18 s, flip angle = 90°, field of view (FOV) = 100 × 100 mm^2^, matrix size = 66 × 66 voxels, slice thickness = 1.9 mm, voxel size = 1.5 × 1.5 × 1.9 mm^3^). On each experiment day, a low-resolution FLASH anatomical scan was acquired with the same geometry as the functional images (TE = 14 ms, TR = 3 s, TA = 2.18 s, FOV = 100 × 100 mm^2^, matrix = 512 × 512 voxels, slice thickness = 1.9 mm, number of averages = 2, flip angle = 150°). For overlaying our functional images, we created a high-resolution anatomical template (0.5 × 0.5 × 0.5 mm^3^ isotropic voxels) by averaging five high-resolution anatomical scans acquired under general anesthesia with an MP-RAGE sequence (TE = 3.0 ms, TR = 2.5 s, flip angle = 8°, FOV = 116 × 96 × 128 mm^3^; matrix = 232 × 192 × 256 voxels).

### Data analysis

For M1, nine EPI runs (180 time points each) were acquired over six sessions. For M2, seven runs were acquired over four sessions. All data analyses were performed using AFNI (Cox, 1996) (http://afni.nimh.nih.gov/afni), FreeSurfer (Dale et al., 1999; Fischl et al., 1999) (http://surfer.nmr.mgh.harvard.edu/), SUMA (http://afni.nimh.nih.gov/) and custom code written in Matlab (MathWorks). Preprocessing involved slice timing correction, motion correction (relative to the run-specific mean GE-EPI), spatial smoothing with a 3.0 mm full width at half-maximum Gaussian kernel, and normalization of the time series at each voxel by its mean. All volumes that had motion values with shifts >0.5 mm and/or rotations >0.5° were excluded from further analyses. Lastly, we performed linear least-squares detrending to remove non-specific variations (i.e., scanner drift). Following preprocessing, data were submitted to generalized linear model analyses. The model included three stimulus-specific regressors and six estimated motion regressors of no interest. For each stimulus category (Env, MC, SMC) we estimated a regressor by convolving a one-parameter gamma distribution estimate of the hemodynamic response function with the square-wave stimulus function. We performed *t*-tests contrasting all sounds vs. baseline (“silence” trials), MC vs. Env, and MC vs. SMC. Finally we co-registered and normalized our functional data to the population-average MRI-based template for rhesus monkeys 112RM-SL (McLaren et al., 2009) and then displayed the results on a semi-inflated cortical surface of the template extracted with Freesurfer and displayed with SUMA to facilitate visualization and identification of cortical activations. The anatomical boundaries described here are based on the macaque brain atlas of Saleem and Logothetis (2012).

To quantify the lateralization of the BOLD response across hemispheres we measured a lateralization index [LI = (*R*_h_ - *L*_h_)/(*R*_h_ + *L*_h_)], where *R*_h_ and *L*_h_ are the mean responses in the right and left hemisphere, respectively. The LI curve analyses ensure that the lateralization effect is not caused by small numbers of highly activated voxels across hemispheres. The LI curves were based on the *t*-values obtained from each contrast condition and were calculated using the LI-toolbox (Wilke and Lidzba, 2007) with the following options: ±5 mm mid-sagittal exclusive mask, clustering with a minimum of 5 voxels and default bootstrapping parameters (min/max sample size 5/10,000 and bootstrapping set to 25% of data). The bootstrapping method calculates 10,000 times LIs using different thresholds ranging from zero until the maximum *t*-value for a specific contrast condition. For each threshold a cut-off mean value is obtained from which a weighted mean (LI-wm) index value can then be calculated (Wilke and Lidzba, 2007). This yields a single value between −1 and 1 indicating right- or left-sided hemisphere dominance.

## Results

Our first goal was to identify brain regions involved in the processing of conspecific vocalizations by the macaque brain. To this end, we collected functional MR images of two monkeys in a horizontal 3-T scanner while stimuli from three different sound categories were presented to the animals. Complex sounds are characterized by having a wide range of spectrotemporal features. While environmental sounds typically contain sharp temporal onsets, monkey vocalizations contain greater modulations in the spectral domain because of the harmonics contained in these sounds. Environmental sounds also carry abstract information about the identity of objects, so a comparison between BOLD responses to monkey vocalizations and environmental sounds is useful in determining brain structures involved in higher-level processing. However, specific spectrotemporal differences exist between these two types of sounds. This can be seen, for instance, in the spectral modulation of monkey vocalizations at approximately 1.5–2 cycles/kHz, which is not present for other sound categories (Figure 1D). Thus, scrambled versions of monkey calls (SMC) were used to further control for the local spectrotemporal features in the vocalizations (see Figure 1C and Material and Methods). Comparison of average modulation spectra between categories showed that SMC were acoustically better matched to MC than Env (correlation coefficient between the modulation spectra: SMC vs. MC: 0.92, Env vs. MC: 0.86; Figure 1D).

**Figure 2:**
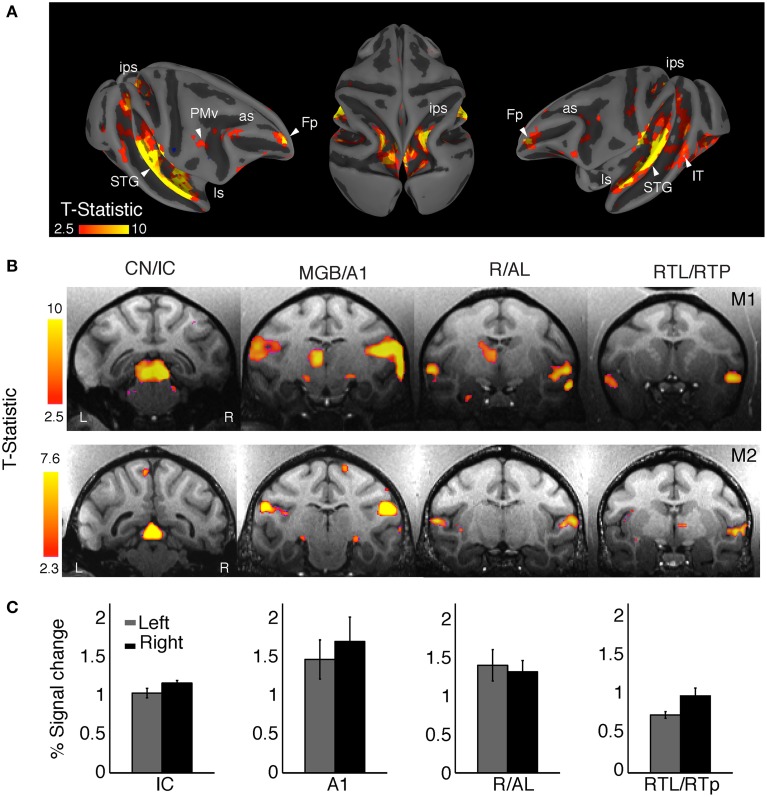
**Mapping auditory and auditory-related regions with complex sounds. (A)** Representative cortical responses from monkey (M1) for all sound conditions combined (q FDR < 0.05, *p* < 10^−2^; cluster size > 10 voxels). The projection onto the semi-inflated surface preserves sulcal and gyral landmarks while allowing visualization inside the intraparietal sulcus (ips) and lateral sulcus (ls). Activation was observed along the auditory ventral stream in the superior temporal gyrus (STG), the superior temporal sulcus (STS), ventral intraparietal area (VIP), and the frontal pole (Fp). Activated dorsal-stream regions included the ips and ventral premotor cortex (PMv). Some active clusters were also observed in the middle temporal area (MT) and the inferior temporal cortex (IT). **(B)** Activation was robust across regions in the ascending auditory pathway of the two monkeys: cochlear nuclei (CN), inferior colliculus (IC), medial geniculate nucleus (MGN), primary auditory cortex (A1), rostral area (R), anterolateral area (AL), lateral rostrotemporal area (RTL), and the rostrotemporal pole region (RTp). (**C**) The average BOLD response for the main auditory activation showed a right-hemisphere bias in both animals (M1, weighted mean = −0.33, M2, weighted mean = −0.66).

Overall, sound stimulation elicited significant BOLD responses compared to silent trials irrespective of auditory stimulus category [q (FDR) < 0.05, *p* < 10^−3^, one-tailed *t*-test, *t* range: 2.3–10, cluster size > 10 voxels] in a broad network of brain regions, including subcortical auditory pathways, classical auditory areas of the superior temporal gyrus (STG), but also regions in parietal and prefrontal cortices (Figure 2). The clusters in Figure 2A highlight the main activation sites on the cortical surface of monkey M1. Figure 2B shows selected coronal slices for both animals (M1 and M2) showing activation in the ascending auditory pathway. These regions include the cochlear nucleus (CN), the inferior colliculus (IC), the medial geniculate nucleus (MGN), the primary auditory cortex (A1), and areas in the anterior superior temporal cortex, including the rostral (R) and anterolateral (AL) areas, the rostrotemporolateral area (RTL), and the rostrotemporal pole (RTp) region.

Activation clusters (averaged across animals and hemispheres) taken from a normalized number of voxels (i.e., equal number of left and right voxels) were found in: IC [*N* = 84 voxels, peak coordinate = (4, −1, 12)]; A1 [*N* = 198 voxels, peak coordinate = (22, 6, 24)]; R/AL [*N* = 131 voxels, peak coordinate = (24, 17, 12)]; and RTL/RTp [*N* = 165 voxels, peak coordinate = (23, 22, 8)].

For both animals we observed a larger amplitude and spatial extent of the BOLD response in the right hemisphere as compared to the left hemisphere (Figure 2B). Activation (percent signal change) in selected clusters for each hemisphere is shown in Figure 2C. We compared the activation between the two hemispheres by calculating a laterality index (LI), with a positive index indicating a left-hemisphere bias and a negative index indicating a right-hemisphere bias. Given the fact that LIs show a threshold dependency (Nagata et al., 2001), we measured LI curves to provide a more comprehensive estimate over a whole range of thresholds (Wilke and Lidzba, 2007). Using this adaptive thresholding approach we found a right-hemisphere bias in the LI curves for general auditory activation (all sounds vs. baseline) in both monkeys (M1, weighted mean = −0.33; M2, weighted mean = −0.66). For higher thresholds, the activation was clustered in primary auditory cortex (A1) of the right hemisphere in each animal.

**Figure 3:**
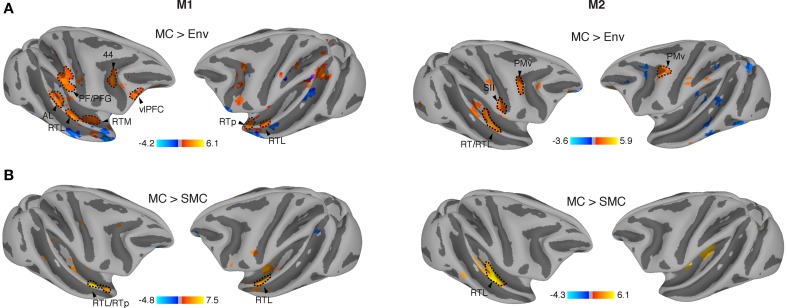
**Regions specifically activated by monkey vocalizations. (A)** Vocalization-sensitive regions obtained from comparison between the effects of monkey calls and environmental sounds. All activation maps were displayed on a semi-flattened surface of the macaque monkey template. Active regions were found in the anterolateral area (AL), lateral rostrotemporal area (RTL), rostrotemporal pole (RTp), secondary somatosensory (SII) cortex, ventral premotor cortex (PMv), ventrolateral prefrontal cortex (vlPFC), and inferior parietal areas (PF and PFG). **(B)** Regions significantly more activated by monkey vocalizations than by scrambled monkey vocalizations include areas in the anterior STG, RTL/RTp. Red/orange: significantly higher activation by MC than by control sounds (SMC or Env); blue: significantly higher activation by SMC or Env than by MC.

Vocalizations are complex naturalistic stimuli that contain behaviorally relevant information. In order to investigate if the auditory system contained representations that are sensitive to this sound category vs. other types of behaviorally relevant complex sounds, we contrasted monkey calls against environmental sounds (see Material and Methods). Environmental sounds also carry abstract information about object identity in their spectrotemporal patterns. We, therefore, also looked for areas showing elevated response to these sounds relative to monkey vocalizations. When correcting for multiple comparisons [q (FDR) < 0.05], no differences were observed for the contrast of MC vs. Env. However, at uncorrected thresholds, we found significantly higher activations by MC as compared to Env in both monkeys across regions in temporal, parietal and prefrontal cortices (M1, *p* < 10^−3^ uncorrected, *t*-value range: −4.2 to 6.1, cluster size > 5 voxels; M2, *p* < 10^−2^ uncorrected, *t*-value range: −3.6 to 5.9, cluster size > 5 voxels) (Figure 3A). Specifically, activations sensitive to MC were found in the anterior STG region, including areas AL and RTp of the rostral belt/parabelt, and further along the auditory ventral stream in ventrolateral prefrontal cortex (vlPFC). In addition, we observed activation patches in the inferior parietal lobule (areas PF/PFG) of the right parietal cortex, and bilaterally inside the inferior branch of the arcuate sulcus, possibly corresponding to Brodmann's area (BA) 44, and posterior to the arcuate sulcus, in a region that is part of ventral premotor cortex (PMv). In addition, we found regions sensitive to environmental sounds (blue) along the superior temporal sulcus (STS) and inferotemporal (IT) cortex. To investigate hemispheric lateralization in the processing of vocalizations, we measured LI curves for this contrast (Mc > Env), finding a slight right hemispheric bias in monkey M1 (weighted mean = −0.19) and a moderate right-hemisphere bias in monkey M2 (weighted mean = −0.42).

In order to determine whether spectrotemporal features alone could have driven the activation in these areas, we further contrasted monkey calls (MC) with scrambled monkey calls (SMC). The results showed similar patterns of MC activation in both monkeys in the RTL region of the aSTG (M1; *p* < 10^−3^ uncorrected, *t*-value range > −4.8 to 7.5, cluster size > 5 voxels and for M2, *p* < 10^−2^ uncorrected, *t*-value range > −4.3 to 6.1, cluster size > 5 voxels) in both monkeys specifically in the RTL region of the aSTG (Figure 3B). In monkey M2, a second region, the middle medial belt (MB), was also more strongly activated by monkey vocalizations than by their scrambled counterparts. The weighted-mean lateralization index (LI) for this contrast (MC > SMc) also showed higher values toward the right hemisphere (M1: weighted mean = −0.34; M2: weighted mean = −0.44). A summary is shown in Table 1.

**Table 1:** **LI-weighted-mean values for the overall sound activation and for each contrast condition**.

	**All > baseline**	**MC > Env**	**MC > SMC**
M1	−0.33	−0.19	−0.34
M2	−0.66	−0.42	−0.44

*Mean lateralization index values (LI-wm) are shown that were obtained from LI curves measured as a function of the statistical threshold (t-value) for the overall auditory activation (all sounds vs. baseline), for the contrast between monkey calls and environmental sounds (MC > Env) and for the contrast between monkey calls and scrambled monkey calls (MC > SMC). A positive index indicates a left-hemisphere bias, while a negative index indicates a right-hemisphere bias. LI-wm values are shown separately for monkeys M1 and M2*.

**Figure 4:**
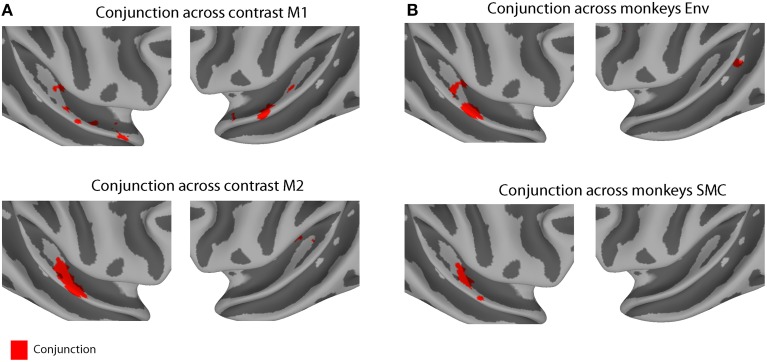
**Conjunction results across contrast conditions and across monkeys. (A)** Conjunction across contrast (MC > Env and MC > SMC) for monkey M1 (*n* voxels = 235, top panel) and for monkey M2 (*n* voxels = 89, lower panel). **(B)** Conjunction across monkeys (M1 and M2) for contrast MC > Env (*n* voxels = 248, top panel) and for contrast MC > SMC (*n* voxels = 58, lower panel). Each individual contrast map was thresholded at *p* < 0.01 (uncorrected). Red region indicates conjunction voxels that were differentially activated for both contrasts in each monkey or in both monkeys for each contrast.

Some differences in the patterns of activity were observed across the two animals. These differences might be explained either by variability across subjects or by differences in attentional state: M1 was significantly engaged in completing the task (>90% success), whereas M2 was scanned passively while holding fixation. To compensate for this variability, we calculated the minimum *t*-statistic (*p* < 0.01 uncorrected) across contrasts in each monkey (a conjunction test) and across monkeys in each contrast (Figure 4). Conjunction across contrasts (MC > Env and MC > SMC) and monkeys (M1 and M2) found a single area in the right hemisphere to be specifically involved across both conjunction analyses, area RTL/RTp (peak coordinate: 24, 17, 12).

## Discussion

Species-specific vocalizations in non-human primates (“monkey calls”) convey important information about affective/emotional states as well as the recognition of objects and individuals (Ghazanfar and Hauser, 1999). We used whole-brain functional magnetic resonance imaging (fMRI) in awake behaving monkeys to examine auditory responses to stimuli from three different sound categories: (a) multiple types of conspecific monkey calls, (b) environmental sounds, and (c) scrambled versions of the same monkey calls largely preserving their local spectrotemporal features.

For all three sound categories combined we found robust BOLD responses along various regions in the ascending auditory pathways (CN, IC, MGB, and A1, Figures 2A,B). These results, using a 3-T scanner without contrast agent, corroborate previous fMRI findings obtained on a 1.5-T magnet with the contrast agent MION, showing activation by complex sounds along the auditory pathway (Joly et al., 2012a). The results further attest to the fact that complex sounds are highly effective for mapping subcortical and cortical auditory structures (Rauschecker et al., 1995; Rauschecker, 1998b; Poremba et al., 2003). Furthermore, our results confirm the general trend of a slight right-hemisphere bias (Table 1) in the processing of complex sounds in the macaque auditory cortex, as measured with fMRI (Petkov et al., 2008; Joly et al., 2012a). Similar results have been found in humans for non-speech voice sounds (Belin et al., 2000).

When we compared activations produced by monkey vocalizations vs. the other two sound categories using a conjunction analysis, we found consistent activations in regions along the anterior STG, in particular in areas AL, RTL and RTp, in both animals (Figure 4). Our results extend previous findings of increased sensitivity to monkey vocalizations in anterior STG regions (Poremba et al., 2003; Petkov et al., 2008; Kikuchi et al., 2010; Joly et al., 2012a,b; Fukushima et al., 2014) by using control stimuli (SMC) that retained the low-level acoustic information of macaque vocalizations and whose acoustic structure was better matched to the vocalizations than the acoustic structure of other complex sounds (Figure 1D). Single-unit studies of the R/AL region have also found increased selectivity either to monkey calls, or to sound categories including vocalizations (Tian et al., 2001; Kuśmierek et al., 2012), consistent with the present results (Figures 3, 4).

Thus, the cortical representation of vocalizations involves an auditory ventral pathway, consisting of a chain of interconnected regions in anterior STG and vlPFC that extract abstract information for the recognition and categorization of vocalizations (Rauschecker, 2012). The rostral belt, parabelt and aSTG send afferent projections into ventrolateral, polar, orbital, and medial regions of the prefrontal cortex (PFC) (Jones and Powell, 1970; Hackett et al., 1999; Romanski et al., 1999; Cavada et al., 2000; Kaas and Hackett, 2000; Hackett, 2011; Yeterian et al., 2012), and together these regions form the ventral cortical stream in audition. Vocalization-sensitive neurons are found along with face-sensitive neurons in the vlPFC (Romanski et al., 2005), allowing these regions to integrate vocalizations with the corresponding facial gestures (Romanski and Goldman-Rakic, 2002; Cohen et al., 2007; Diehl and Romanski, 2014). The PFC is involved in higher-level integrative processes for the cognitive control of vocalizations as well as in the interpretation of semantic content in vocalizations (Romanski and Averbeck, 2009). The activation patterns observed in PFC (Figure 3A) could represent categorical or affective information reflected in the vocalizations. Further imaging studies and multivariate analyses comparing multiple vocalization types might elucidate the differential contribution of each subregion of the PFC.

Our stimuli also activated higher-level visual areas, such as the middle temporal (MT) and inferior temporal areas (IT). These areas are known to be involved in the processing of visual motion (Maunsell and Van Essen, 1983) and in object perception (including faces), respectively (Tsao et al., 2006; Ku et al., 2011). Their activation by purely auditory stimuli raises interesting questions regarding their possible role in the multisensory processing of dynamic audio-visual stimuli, such as facial expressions that naturally occur in conjunction with vocalizations and/or motion of the face (Furl et al., 2012; Polosecki et al., 2013; Perrodin et al., 2014). However, to answer these questions more definitively, further imaging experiments utilizing dynamic audio-visual stimuli would be necessary. Such studies could enlighten us on how auditory information combines with visual information in both the ventral and dorsal pathways building multimodal representations from dynamic facial expressions combined with vocalizations (Ghazanfar and Logothetis, 2003).

When we contrasted monkey calls to environmental sounds, we also found differential activation in regions PF/PFG (area 7b) (Pandya and Seltzer, 1982; Rozzi et al., 2006) of the inferior parietal lobule (IPL), in addition to the well-known regions in the STG sensitive to monkey vocalizations. Parietal regions inside the intraparietal sulcus (IPS) have been known to receive auditory projections (Lewis and Van Essen, 2000) and to contain neurons that respond to auditory and multimodal stimuli (Stricanne et al., 1996; Bushara et al., 1999; Grunewald et al., 1999; Cohen and Andersen, 2000; Cohen, 2009), but the role of these regions has traditionally been assumed to lie in spatial processing and control of eye movements.

Similarly, we found an engagement of the ventral premotor cortex (PMv) in the processing of monkey vocalizations (Figure 3A). This region has previously been thought to be involved in the processing of the location (but not quality) of nearby sounds (Graziano et al., 1999). Surprisingly, when we compared the effects of vocalizations (MC) against vocalizations that were scrambled in both the spectral and temporal domains (SMC), we did not observe greater activation in parietal or prefrontal areas for MC, suggesting that the scrambled versions of the MC evoked the same amount of activity in these regions. Similar results were obtained by Joly et al. (2012b) with temporally scrambled vocalizations activating large regions of premotor and parietal cortices. Ventral premotor cortex (PMv) has also been implicated in the initiation of vocalizations in the macaque monkey (Hage and Nieder, 2013). It appears possible, therefore, that the same neurons are the source of an efference copy signal (Kauramäki et al., 2010), which is responsible for the suppression of auditory cortex during self-initiated vocalizations (Eliades and Wang, 2003). More generally, they could be part of an audio-motor network connecting perception and production of sounds (Rauschecker and Scott, 2009; Rauschecker, 2011).

## Author contributions

MO co-designed the study, trained the animals, programmed stimulus presentation, acquired part of the data, conducted most analyses, and co-wrote the manuscript. PK programmed the behavioral task and participated in writing the manuscript. DA trained the animals and acquired part of the data. ID generated the scrambled stimuli and acquired part of the data. FA contributed with data analyses and participated in writing the manuscript. GK, interpreted data and participated in writing the manuscript. MS, IJ, and JR co-designed the study and participated in writing the manuscript.

### Conflict of interest statement

The authors declare that the research was conducted in the absence of any commercial or financial relationships that could be construed as a potential conflict of interest.
